# “Argue With Me”: A Method for Developing Argument Skills

**DOI:** 10.3389/fpsyg.2021.631203

**Published:** 2021-03-04

**Authors:** Kalypso Iordanou, Chrysi Rapanta

**Affiliations:** ^1^School of Sciences, University of Central Lancashire, Larnaka, Cyprus; ^2^Faculty of Social Sciences and Humanities, Universidade NOVA de Lisboa, Lisbon, Portugal

**Keywords:** argumentation, literacy, epistemology, critical thinking, writing, curriculum, citizenship

## Abstract

Philosophers, psychologists, and educators all acknowledge the need to support individuals to develop argument skills. Less clear is *how* to do so. Here, we examine a particular program, the “Argue with Me” dialogue-based pedagogical approach, having this objective. Reviewing approximately 30 studies that have used the “Argue with Me” (AWM) method with students of different backgrounds and educational levels—primary, middle, high school, and university—across five different countries, we examine its strengths and limitations in terms of what develops and how this development occurs. Dense engagement in goal-based activities involving extended dialogic practice and reflection is shown to be effective in fostering argument skills and dispositions. Studies examining the mechanisms of such development identify the role of *meta-level* understanding regarding the purpose of argument. This understanding is epistemological in nature and supports the development of dialogic skills at the strategic level. In addition to examining the AWM method as a means for supporting the development of argument skills, this review examines how empirical research employing the method in varying contexts provides insights into the nature of argument skills and their development, as well as the relations between argument skills and other skills or forms of understanding. For instance, we examine how studies employing the AWM method answer questions such as “How general or content-specific are argument skills?” or “How do dialogic argument and individual written or spoken argument connect as they develop?” We address these questions by examining evidence regarding the transfer of gains across topics, domains, and individual vs. dialogic modes of expression. Finally, the pedagogical implications of the “Argue with Me” approach are discussed, especially with regard to its potential both as a stand-alone method for developing argument skills and integrated into traditional literacy and social studies curricula.

## Introduction

The topic of argument skills is as old as the existence of human thought about reasoning, which came to light with some early philosophers’ work in ancient Greece and Rome, with Aristotle and Cicero the most representative examples. Among other contributions, Aristotle distinguished between the different types of common places (*topoi*) for logical premises to be drawn on, while Cicero highlighted the indispensable connection between the logical construction of arguments (*invenire*) and their rhetorical elaboration (*orare*). The art of dialectics (διαλεκ*τ*ικ*ń*) was born and, along with it, the need for methods to ensure the construction of better (more logical *and* more persuasive) arguments. The need to gain an understanding of the links between logic, rhetoric, reasoning, and cognitive development has been a pressing one since ancient times.

In recent years, there is an increased interest in research on argumentation (Nussbaum, 2008; Garcia-Mila et al., 2013; Reznitskaya and Gregory, 2013; Murphy et al., 2018; Resnick et al., 2018; Larrain et al., 2020). In this article, we focus on the ideas and research data reflected in a particular line of research, the *Argue with Me* (AWM) approach, to developing argument skills and dispositions developed by Kuhn and colleagues (Kuhn et al., 2016a). The need for this theoretical and empirical overview emerges from a current lack of a qualitative synthesis explicitly focusing on studies that have implemented this innovative pedagogical method, on one hand, and an increasing evidence that this method works when it comes to argument skills’ development, on the other. One aim was to add to the understanding of what develops and the mechanisms that support this development. In addition, we aimed to identify pedagogical implications as well as directions for current and future research into the still underexplored paths of argument skill development.

The article is structured as follows. Firstly, a synthesis of the major theoretical assumption behind Kuhn’s dialogical argumentation pedagogical method will be presented. After this theoretical framing, we will pass to the empirical part of our review, making explicit its concrete questions that guided our analysis of approximately 30 studies implementing the AWM curriculum until today. Conclusions and recommendation emerging from this analysis will be presented at the end.

## Theoretical Assumptions of the Argue With me Curriculum

Influenced by the twentieth-century psychologist Billig (1987) and the argumentation theorist Douglas Walton (1989), Kuhn makes the following series of claims.

### Everyday Thinking Is by Nature Argumentative

Rooted in the early origins of informal reasoning, as a form of reasoning aiming at dealing with everyday problems and decisions, there lies the idea that the greatest part of human thinking is about ill-defined issues, and as such, a kind of thinking appropriate for resolving those is necessary. This thinking must focus on assessing, weighing, and using the information available as relevant and adequate to support one’s position leaning toward an action or a belief. Many have named this thinking critical thinking, with argument construction and evaluation being one of its main goals. Kuhn, however, takes a step further: combining critical thinking and informal reasoning theories, she claims that a view of thinking as argumentation is necessary, one that goes beyond a thinking performance using valid arguments and sees argumentation as a main practice path toward the development of skills necessary for citizenship in a democratic society. The idea of argument *as* thinking (Kuhn, 1992) gained further insights later as a more comprehensive view of a dual relationship between argument as a critical thinking practice leading to more argumentative thinking products translated into better (more reasoned, sophisticated, and weighed) decisions and proposed solutions to everyday problems. Her most recent book titled *Building our Best Future: Thinking Critically about Ourselves and Our World* (Kuhn, 2018b), written directly to adolescents, explicitly focuses on the critical thinking practice of argumentative reasoning aiming at the informed decision-making and problem-solving practices of adolescents.

### The Argumentative Nature of Thinking Needs Dialogue

Kuhn (1992) made the distinction between a rhetorical and a dialogic argument, the former referring to “a course of reasoning aimed at demonstrating the truth or falsehood of something” (American Heritage Dictionary, 1981, cited in Kuhn, 1992, p. 157) and the latter referring to an argument in course, meaning between at least two people. The same distinction is described by O’Keefe (1992) with the terms *argument1* and *argument2* types of argument and, later (Johnson, 2002), as *argument-as-product* (i.e., something that a person makes) and *argument-as-process* (i.e., something that a person engages in). Moreover, from an informal logic point of view, the argument structures (i.e., a set of propositions with certain characteristics) manifested within arguments-as-products, or rhetorical arguments, presuppose the process of argumentation within which they are produced (Johnson, 2002), i.e., the argument-as-process in which they emerge. In other words, rhetorical arguments are necessarily dialogic, in a twofold sense: (a) as part of their structure, as any reasoned argument at least implies an opposite or alternative viewpoint; otherwise, it would be a mere inference or reasoning, without an argument notion expressed within (for a distinction between argument and reasoning, see Walton, 1990), and (b) as part of their function, as an argument cannot be identified and/or assessed out of its context, and this context is necessarily communicative even when the argument is expressed intra-psychologically and not inter-psychologically, as for example in a speech/lecture or even in written discourse. These two assumptions have formed the basis of Kuhn’s most recent thought, especially when it comes to the development of the idea that critical thinking is necessarily dialogic and manifested through the practice of argumentation (Kuhn, 2018a, 2019).

### Dialogue to Be Nurtured Needs Intentional Thinking

If argument is necessarily dialogic, as explained above, then what characteristics does dialogue need to have in order to be productive? Although Kuhn and her colleagues do not use the term “productive” as a characteristic of educational dialogue as other scholars do (see for example Resnick et al., 2010), they imply that dialogue is productive when it leads to the development of argument skills, in oral or written discourse form. In other words, the productivity of dialogue is not a characteristic to be judged *a priori* or in the course of dialogue itself, for example when certain norms of dialogic behavior are met, as in the case of Mercer’s (1995) exploratory talk, but only *a posteriori*, after the arguments produced are assessed as being reasoned and of a certain dialogic quality (i.e., dual, integrated, etc., in the case of written arguments; transactive, dialectical, etc., in the case of oral argument moves). However, what can and should be done *a priori*, in Kuhn’s perspective, is the design of the argument learning environment on the basis of one main assumption: the goal-orientedness of the activities through which the gradual immersion in the argument-as-process nature and objectives is achieved. Although many educational researchers (e.g., Nussbaum et al., 2005; Nussbaum, 2008; Garcia-Mila et al., 2013) have suggested that the goal of argumentation made explicit to students must be one of collaboratively reaching a consensus, rather than convincing the other party (i.e., a “win–win” rather than a “win–lose” situation), Kuhn has opted for maintaining the dialectical nature of persuasive argumentation while at the same time framing it as a collaborative activity. In Kuhn’s account, an argument is like a ball that needs to be successfully hit from one side to the other in order for the game (argument-as-process) to be on. At the end, the party that has made the best hits wins. This view of persuasive argumentation as a participation in a collaborative, yet competitive, game has several pedagogical implications: (a) that each party needs to have several instances of intra-team collaboration in order for their hits to be as successful as possible; (b) that each party needs to anticipate the other party’s hits in order to receive them and respond to them adequately; and (c) that both parties are interested in the game going on, therefore receiving and replying to the other party’s hits throughout the course of the game. From an argumentation theory point of view (Walton, 1989), the several “hits” aim at increasing the strength of the argument at hand, when it comes to one’s own arguments, or decreasing it, when it comes to responding, implicitly or explicitly, to the other side. Therefore, the goal of argumentation is dual: on one hand, one must search to “secure commitments from the opponent that can be used to support one’s own argument”; on the other hand, (s)he must “undermine the opponent’s position by identifying and challenging weaknesses in his or her argument” (Kuhn and Udell, 2003, p. 1246).

The preceding ideas gave gradual rise to the development of a method aiming to support individuals’ argument skills, called “Argue with Me” (AWM). The method, first fully implemented by Kuhn et al. (2008), with earlier versions by Felton (2004) and Udell (2007), involves extensive practice in argumentation and reflection in the context of a goal-based activity that keeps participants’ motivation high. Since 2008, the method has been implemented in many schools, with consistent findings. Currently, there are 29 empirical papers describing studies where the AWM method has been implemented. The duration of the AWM intervention ranged from as short as six intervention hours over 2 days (Iordanou et al., 2019) to longitudinal twice-weekly implementations, up to 3 years (Crowell and Kuhn, 2014).

**FIGURE 1:**
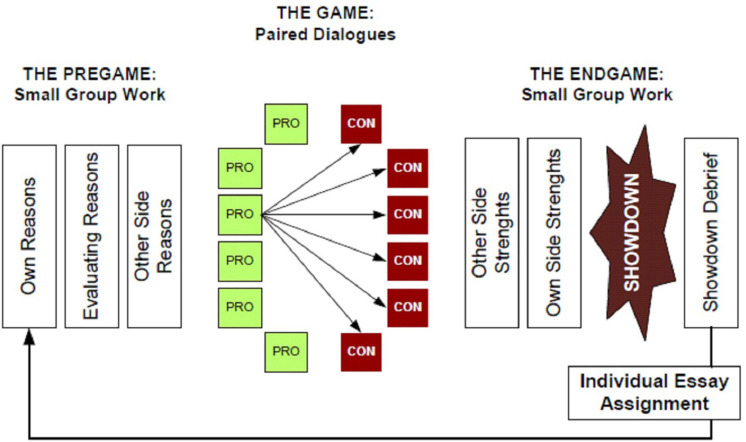
The AWM structure.

AWM is structured into three main phases—Pregame, Game, and Endgame—with different cognitive and dialogic objectives in each. Table 1 presents a summary of the activities and specific cognitive and dialogic goals at each phase, while Figure 1 depicts the sequence.

**TABLE 1:** Cognitive and dialogue objectives of the three phases of the “Argue with Me” (AWM) method and its main activities (adapted from Kuhn et al., 2016a).

Cognitive objectives	Dialogue objectives	Main activities
**Pregame**		
Understand that reasons underlie opinions, different reasons exist for the same opinion, and some reasons are better than others.	Elaborate argument blocks using reasons (evidence) to support opinions.	Small-group brainstorming, one-to-one ideas’ elaboration and synthesis, and small-group analytical discussion
**Game**		
Understand that opponents have reasons too, reasons can be countered, and counters to reasons can be rebutted.	Generate counterarguments to other’s reasons and rebuttals to one’s counterarguments.	Dyadic written or semi-oral discourse, pair-to-pair confrontation, and within-pair reflection
**Endgame**		
Understand that the same information can be used as evidence to support or weaken different claims.	Weigh opposing positions in a framework of alternatives and evidence.	Small-group reflection, one-to-one debate, whole-class reflection, and individual writing
		

The goal of this paper was to provide an overview of the major outcomes of the 29 empirical studies that have applied the AWM method thus far.

## What Develops?

The development of argument skill is multifaceted and gradual (Kuhn et al., 2013). The work by Kuhn and colleagues implementing the AWM method in different contexts and with the manipulation of different variables sheds light on the complex nature of argument skill development and its underlying mechanisms. In this section, we seek to identify the particular gains in argumentive competence empirically related to the AWM implementation, as well as the specific characteristics of the dialogue-based method that may promote one gain or another. Table 3 shows the gains of engagement in the AWM curriculum in all the studies that the AWM method has been implemented.

Argumentive reasoning development encompasses two main manifestations: the first is related to the *production of valid arguments*, either individually or interactively, while the second is related to *relevant forms of participation* in argumentive dialogue. These two manifestations, previously described in the *Introduction* as argument-as-product and argument-as-process, will now be given a closer look in terms of their specific development reported in the AWM-related empirical research. We then proceed from skill development to gains in content knowledge.

### Constructing Valid Arguments

A valid argument can be represented by an idea unit containing a claim supported by a piece of information supporting that claim. This idea of *functional support* is highly important as it represents informal logic criteria, such as sufficiency and acceptability, as described by Blair and Johnson (1987). For an idea to be sufficiently supported, the selected information must be linked to it clearly and explicitly enough for the logical relation between the two to be revealed. According to Toulmin (1958), this kind of logical relation can be of two types: (a) an explanation of how what is claimed to be a support naturally links to the claim itself (*warrant* in Toulminian terms) or (b) a justification of why this specific linkage between claim and support must be considered as evidence that the claim is true (*backing* in Toulminian terms). Without establishing connections neither with the informal logic criteria nor with Toulmin’s theoretical contribution of a valid argument structure, Kuhn and colleagues seem to be claiming something very similar with their simplified notion of a functional unit: for a claim to be characterized as evidence-based, the connection between the alleged evidence and the claim must be explicit, and the evidence must be accurate (not “mischaracterized;” Shi et al., 2019, p. 118), meaning that the original meaning and context of the information serving as evidence must not be altered, as in the case of falsification of information in social media fake news and stories. Moreover, the use of factual information as evidence must be done in a way that is logically acceptable, meaning not violating the standards of soundness and cohesion (Blair and Johnson, 1987). This, in Kuhn’s terms (Kuhn et al., 2013), corresponds to a commitment to “accountable talk” (Michaels et al., 2008), implying that the claims put forward, both proposing and supporting ideas, are based on shared standards of reasoning and knowledge (otherwise the use of information would be fallacious or paralogical; see Rapanta and Walton, 2016). All of the above aspects constitute what can be called evidence-based reasoning. What makes Kuhn’s contribution unique, however, is her conceptualization of evidence not as a static entity with an *a priori* given status but as a functional unit itself, subset to pragmatic modifications according to its use. Information becomes evidence when employed in relation to a claim.

Pragmatic modifications can be of four main types, namely data used to support one’s own view, data used to support the other party’s view, data used to weaken the other side’s view, and data used to weaken one’s own view. These different functional uses of information as evidence give validity to an argument, as it is not the evidence itself that is more valid than another. This view explains also the fact that, for Kuhn (see Kuhn and Moore, 2015; Kuhn, 2015), two types of evidence (or better said *information used as evidence*) are possible: the shared, i.e., based on a set of information made available to students, and the personal, i.e., generated from the students’ own personal knowledge.^1^ For both types, the same criteria of functionality apply. Table 2 illustrates the identification of functional units in an 11 year-old female Portuguese student essay, after she participated in the AWM curriculum, on the topic of whether we should immediately end the use of plastic or not.

**TABLE 2:** Rationale behind the identification of functional units (i.e., valid arguments) in a student’s essay.

Text	Functional (FU) and non-functional (NF) unit identification with explanation
“I think we should ban the plastic because it kills many animals, it pollutes the environment, causes fires, and it causes the global warming.”	*This sequencing of reasons without a further connection to the claim would have been coded as NF if the student did not continue to explain each one subsequently. Therefore, we assume it is just an introduction to her reasoning that follows.*
“There are many alternatives to replace it, for example glass, metal, bambu?”	*The existence of alternatives to the plastic was among the information provided to students in a Q&A format. Therefore, we consider this as a shared FU.*
“Some time ago, I saw a documentary with my mum that the straws (among other plastics) because they are light they fly with the wind, they go to the sea, and the penguins (among other animals) were eating them thinking that they were food and it stayed in their bellies it was giving them the feeling that they were full and they didn’t manage to eat.”	*Here, the student presents a piece of personal knowledge as evidence for her claim previously made in her short introduction (i.e., it kills many animals). Therefore, we consider this as a personal FU.*
“And the animals are food of other animals.”	*This further reasoning misses an important link (i.e., if some animals die, more animals would do so) to be considered as FU. Therefore, it is NF.*

A necessary counterpart of evidence-based reasoning is the skill of antilogos, namely the ability to identify contrary commonplaces to one’s own assumptions and positions, which may lead to totally different or even oppositional claims and positions (Billig, 1987). Coherent to the idea that evidence relates to one’s own understanding of something that “if found and correctly understood, could change one’s knowledge, one’s beliefs, concerning some matter” (Buckland, 1991, p. 353), reasoning deriving from evidence may lead toward one conclusion or another based on its interpretation and use each time. This is why for Kuhn and colleagues, any construction of a valid argument implies coordination between claim and evidence (Kuhn and Moore, 2015; Hemberger et al., 2017). Such coordination, if successful, allows dialogue participants not only to argue against an opponent but also to adequately reply to his/her counterarguments by means of a rebuttal (Kuhn et al., 2008). Both counterarguments and rebuttals, and the different strategies used to express them in a dialogue, are manifestations of the antilogos skill, which in turn is an essential ingredient of critical thinking and argumentation (Walton, 1989).

Addressing the question of what aspects of evidence-based reasoning and antilogos skills are promoted as result of the AWM curriculum, a common finding across the empirical studies reviewed is that, following participation, students more often search for and use evidence in their efforts primarily to support their own and undermine the other’s position, but also to a lesser extent to address evidence and arguments incongruent with their own position (Kuhn et al., 2008, 2016b; Iordanou and Constantinou, 2015; Kuhn and Moore, 2015; Hemberger et al., 2017; Shi, 2019; Shi et al., 2019; Iordanou and Kuhn, 2020), and more efficiently (Iordanou, 2010, 2013; Crowell and Kuhn, 2014; Kuhn and Crowell, 2011; Iordanou and Constantinou, 2014; Papathomas and Kuhn, 2017; Matos, 2021). This behavioral, as contrasted to the epistemological (discussed below), increased facility with what counts as evidence and how it can serve one’s argumentive reasoning is a central benefit of the AWM curriculum.

But what are the particular aspects of the dialogue-based method that render these gains possible? The AWM method is a complex, multicomponent intervention, and specific experimental dissection is required to isolate its effective components. Some of the reviewed studies suggest the *dyadic intense dialogic interaction* taking place during the Game, and characteristics of it thereof, as a major factor leading to argument gains, whereas others focus on particular elements of the AWM method to address this question, examining for example the *role of reflective activities* or the *type and order of relevant information* made available to the students during Pregame and Game. Each of these components is examined below.

### Dyadic Intense Dialogic Interaction

The idea that dyadic argumentation is a means of cognitive engagement is rooted in the Vygotskian tradition highlighting the complementarity of social and internal thinking. A pioneering study by Kuhn et al. (1997) not only supports this view but also highlights the types of cognitive gains the dialogical argumentive engagement with a peer may lead to. This study, which was a predecessor of the Game phase of the AWM method, showed that, after their systematic immersion in dyadic argumentation over 5 weeks, both adolescents and adults showed evidence of reasoning improvement. Gains included shift from one-sided to two-sided arguments, arguments based within an alternatives framework, and metacognitive awareness of the coexistence of multiple views. The study additionally suggested how different forms of dialogic interaction contributed to different forms of change.

Subsequent studies implementing the AWM method further confirmed the role of intensive dialogic engagement in argument skill development. For example, Iordanou and Constantinou (2015) compared students who engaged in the AWM method in the context of a web-based learning environment, SOCRATES, which included a rich database on the topic of climate change. Eleventh graders serving in the experimental condition engaged in the AWM method, while a group of peers studied the same database for the same amount of time but did not engage in an argumentive discourse activity. Students in the experimental condition increased use of evidence in their dialogues, used more evidence that functioned to weaken the opponents’ claims, and used evidence more accurately. Iordanou and Kuhn (2020) examined which forms of dyadic interaction are more beneficial, comparing individuals who engage in discourse with peers who hold an *opposing* view with individuals who engaged in discourse with peers who hold the *same* positions as themselves. Young adolescents were given access to identical relevant evidence and engaged in dialogues on a physical science topic. In the experimental condition, electronic dialogues were conducted with a series of peers who held an opposing view; in the control condition, dialogues were confined to same-side peers. The results showed differences in the extent and types of functional evidence-based argumentive idea units in individual final essays on both the intervention and a transfer topic, favoring the experimental condition. Extension of the study longitudinally to a second year with a new topic showed continued gains and condition differences, with the experimental group surpassing the control group. This study further suggests that adversarial argumentation, employing the aim to persuade, is a more productive means, compared to coalescent or collaborative argumentation, to support the development of argument skills. Matos (2021) showed that incorporating a collaborative writing activity with opposing side pairs in the AWM method yielded greater gains in terms of using evidence and integrating belief-incongruent and belief-congruent statements compared to engagement in the AWM method without this additional element.

Another group of studies aimed at examining whether the skills of the partner that one collaborates with while engaging in the AWM method function as a further scaffold for the observed individual argument gains. Zillmer and Kuhn (2018) found that peer collaboration, that is, having same-side peers to collaborate while they engage in discourse with other pairs holding opposing position, supported individuals’ argumentation skills. The benefits of collaboration extended to equal—as well as unequal—ability peers, a condition not emphasized in Vygotsky’s writing. *Same*-ability partners could flexibly scaffold one another with metacognitive support, taking on the role of support provider and support recipient interchangeably and as needed.

### Reflective Activities

Another core element of the AWM method is engagement in reflective activities about one’s own argumentation. Significant advancements in students’ use of evidence were observed after their engagement in reflective activities that prompted them to reflect on the use of evidence in their arguments, when students’ progress was examined using a microgenetic method (see Iordanou and Constantinou, 2015). When an additional reflective activity about evidence was included in the AWM method, it proved more effective in promoting students’ use of both types of evidence (congruent and incongruent) and therefore a superior argument performance compared to the AWM method without this additional reflective activity or to a regular school curriculum (Shi, 2019). This was particularly evident in the construction of “However” arguments, i.e., pairs of two units with one unit supporting the opposing side or weakening one’s own side immediately accompanied by a unit supporting one’s own or weakening the other side. In their essays on the last intervention topic (topic 3) in Shi’s study, the majority of students successfully coordinated claims and (incongruent) evidence at least once. Iordanou (submitted) directly investigated the role of reflection in the AWM method by comparing a group who engaged in the AWM curriculum with another one that engaged in the AWM method but not in reflective activities. The results similarly showed that the condition that engaged in both reflective and dialogic activities outperformed the condition that engaged only in dialogic activities.

### Use of Scaffold Prompts

Some of the reviewed studies used prompts for reflection or to encourage the use of evidence. Incorporating scaffold prompts that exemplified functions of evidence in relation to a claim accelerated the prevalence of evidence-based claims in essays of low-performing middle schoolers compared to participants in the same year-long dialogue-based intervention who received no or a limited form of evidence prompts (Hemberger et al., 2017) or compared to participants who engaged in their regular school curriculum (Shi et al., 2019). Iordanou et al. (2019) gave particular attention to the specific types of prompts accompanying the use of questions and answers (Q&As) provided to students. In one experimental condition, the standard prompt “Try to use this information in your arguments” was used, whereas for the second experimental condition, the subtracted prompt “Here’s some information about the topic” was used. They found no significant difference between these conditions; rather, the gradual presentation of evidence both congruent and incongruent to one’s own position was the condition that had some significant impact on students’ performance. This mixed evidence presentation was further accompanied by a prompt of the type “Not all of the evidence is going to support your side; if it doesn’t, see if you can deal with it.” This prompt to consider incongruent evidence showed the greatest effect in furthering mastery of a critical argument skill—to acknowledge and address, rather than ignore, evidence that counters one’s favored position.

### Type and Order of Relevant Information (Evidence) Made Available to Students

Hemberger et al. (2017) tested the hypothesis of whether the type of information presented to students in a Q&A format had an impact on the type and quality of arguments produced. The results showed that in topic 1, the students who received pieces of evidence supporting “own side” only did better than the students who received multiple kinds of evidence—supporting own position, weakening other position, supporting other position, and weakening own position—in terms of producing functional (evidence-based) statements, reflecting the easier task they had of using only supporting evidence. However, in the subsequent topics, the latter group, who received multiple kinds of evidence, surpassed the first one, who received only “own side” evidence, in the frequency of evidence-based statements despite their more challenging task of using multiple kinds of evidence. Students who were not given any evidence showed relatively little evidence use, of the self-generated (from the individual’s personal knowledge) type, only in the later topics.

Iordanou et al. (2019) compared the traditional information text format of presenting relevant knowledge to students with the intentionally structured Q&As gradually provided during the AWM Pregame and Game phases. Although students in both groups became successful in making functional use of the evidence available to them, greater improvement was observed in the Q&A condition. This improvement was significant also in the use of the “weaken other” type of evidence, which is noteworthy given the fact that the use of evidence to weaken a claim is more challenging than the use of evidence to support a claim (Hemberger et al., 2017). A subsequent study (also reported in Iordanou et al., 2019) focused on the order of the presented Q&As, i.e., whether the facilitative order previously presented (Hemberger et al., 2017) made a difference. They found that although the order did not play a significant role, the gradual presentation of evidence that is both congruent and incongruent with one’s own position did indeed result in greater student gains, as further explained in the next section.

A subsequent study by Shi (2019) focused on the prompted mixed evidence condition. Instead of presenting the congruent and incongruent evidence all at once, Shi (2019) opted for sharing one Q&A piece of evidence at a time, for each Game dialogue session. In addition, students were also encouraged to formulate questions they wished to have answered, which the research team provided at a subsequent session. This addition increased the use of claim-congruent evidence (“support own” and “weaken other”).

### Participating in Argumentive Discourse

Relevance as a characteristic of discourse participation implies more than that contributions are on the topic and coherent. In skilled argumentive dialogue, an important role is played by *structural* relevance, i.e., how the argument components logically interrelate (Macagno, 2016). Another aspect of relevance refers to consistency with *pragmatic* function, i.e., the purpose of the dialogue and the different forms it may take throughout interaction (Macagno, 2016, 2019). In skill development terms, the structural form of relevant participation is captured in strategic and metacognitive skills, whereas pragmatic relevance is expressed in terms of metastrategic and epistemological awareness. Both are of core importance within the AWM curriculum and are manifested in particular gains, as elaborated below.

A recent review by Iordanou et al. (2016) shows how argumentive reasoning, including both the construction and evaluation of arguments, and epistemic cognition, i.e., “an umbrella term encompassing all kinds of explicit or tacit cognitions related to epistemic or epistemological matters” (Chinn et al., 2011, p. 141), are intertwined. In this section, we show how this interrelation is manifested and achieved within the AWM curriculum, and in particular through components that promote students’ metacognitive and epistemological development.

According to Moshman (2015), epistemic cognition can be both domain-specific and domain-general, and the same applies for epistemic development, which is the progress in epistemic cognition. Similarly, for Kuhn (1999), there are three types of meta-knowing, namely the metacognitive, the metastrategic, and the epistemological knowing. The differences between the three are subtle but important to consider, especially when it comes to distinguishing the metacognitive from the other two types. For Kuhn (1999, 2001) and others (e.g., Swanson, 1990; Schraw, 1998), metacognition has a declarative component, namely a verbal aspect that directly contributes to one’s (self-)regulation of knowing. Therefore, when we talk about metacognitive skills, we assume that there is an explicitly verbal behavior that shows that an individual is aware of his/her own knowledge. Manifestations of such verbal behavior occur when participants label the argument components (e.g., “here are my counterarguments against your position,” “that’s my evidence against yours,” etc.) or when they talk about their own understanding of the reasons and evidence to support those (e.g., “this evidence supports that reason,” “I don’t know how to use this information to counter the other party,” etc.). However, very often, these explicit verbal expressions do not take place, and self-regulation of knowledge is implicit and occurs together with other verbal behaviors that imply such self-regulation. This is so in the case of strategic use of evidence or of language to defend one’s own position. Examples of these strategic metacognitive skills include the use of discursive strategies to defend one’s own or counter another’s position, which can vary from a simple clarification of one’s own premises to more advanced counterargument and rebuttal strategies (e.g., undermining).

Empirical research applying the AWM curriculum has shown that students’ immersion in a sequence of dialogic activities with a different setting and goal has been proven effective in the use and development of both oral and written strategic argumentive discourse.^2^ For example, the structured engagement in pair-to-pair dialogue, often mediated by electronic means, has been shown effective in immediate (e.g., Mayweg-Paus et al., 2015; Papathomas and Kuhn, 2017) and gradual (Kuhn et al., 2008, 2013; Kuhn and Crowell, 2011; Crowell and Kuhn, 2014; Iordanou et al., 2019; Iordanou, submitted) advancement in students’ counterargument and rebuttal strategies.

When it comes to writing, extensive research (Crowell and Kuhn, 2014; Kuhn et al., 2016a; Hemberger et al., 2017; Iordanou et al., 2019; Iordanou, in press; Shi et al., 2019) involving implementation of the AWM curriculum has shown that dense engagement in oral argumentive interaction benefits the construction of two-sided texts. Such two-sidedness is also reflected in the formation of either a dual or the more advanced integrated perspective. Texts adopting a dual perspective recognize at least once the existence of a contrary or alternative perspective to one’s own, whereas integrated argumentive texts include at least one sequence of adjacent statements of opposing perspectives. Emergence of this “However” argumentive structure (Kuhn et al., 2016b) has been a frequent marker of argumentive reasoning gains.

Another group of epistemic skills, more representative of the “meta” aspects of reasoning, are the so-called metastrategic skills. These refer to: (a) meta-level awareness of the goals of argumentive discourse and the strategies used to achieve them (Kuhn and Udell, 2003; Iordanou and Constantinou, 2014), also called metastrategic awareness (Shi et al., 2019), and (b) epistemological understanding of what constitute acceptable claims, what are acceptable forms to advance those in discourse, and what are the dialogue norms that need to be respected for this to take place (Kuhn et al., 2013; Kuhn and Zillmer, 2015). Empirical research implementing the AWM curriculum has shown that participants develop their epistemic understandings regarding argumentation (Kuhn et al., 2008, 2013; Iordanou, submitted) during their participation, but also their more general epistemological understanding of what is knowledge and how one knows (Iordanou, 2010, 2016; Kuhn et al., 2013; Zavala and Kuhn, 2017; Shi, 2020b).

### Acquiring Knowledge

The “learning to argue” and “arguing to learn” distinction (Von Aufschnaiter et al., 2008; Muller-Mirza and Perret-Clermont, 2009) implies that any intervention primarily aiming to develop argument reasoning gains belongs to the former, while any study primarily seeking knowledge acquisition gains belongs to the latter. The AWM method is representative of a method designed with argument reasoning gains as a primary objective, but content knowledge gains have also been significantly observed, showing that learning-to-argue and arguing-to-learn objectives can be the result of engagement in a single curriculum (Iordanou et al., 2019).

For example, Rapanta (submitted) examined the implementation of the AWM method across four different subject areas by middle-grade teachers in Portugal after a 12 h professional development training focusing on the structure, goals, and activities of the method. As the method was adapted and integrated as part of the curriculum in history, language, citizenship education, and science, the goal of students also achieving content-related gains was explicit. In total, 145 adolescents ranging from the seventh to the 10th grades significantly improved their written answers to an open-ended test question in their corresponding subject areas. This question, chosen by the teachers, was used twice as a pre/post-test assessment and was unrelated to the topics of the intervention. This result, further supported in teacher interviews, shows that argument knowledge construction goes hand in hand with content knowledge construction, as reasoning and knowledge are highly interconnected with one “serving” the other (Iordanou et al., 2016).

In another study, Iordanou et al. (2019) examined the effectiveness of the AWM curriculum in fostering middle-school students’ knowledge acquisition as well as dialogic and written argumentation skills with respect to a content-rich, socially significant topic. The results of two studies, one involving a physical science topic (study I; Iordanou et al., 2019) and the other a social topic (study II; Iordanou et al., 2019), showed that a single intervention could meet both objectives—“learning to argue” and “arguing to learn.” The findings of Iordanou et al. (2019) showing argumentation skill and knowledge gains in the context of a single curriculum have been replicated later by other researchers (Larrain et al., 2020).

## Mechanisms of Argument Skill Development

The strength and value of instructional approaches lie in their ability to promote knowledge or skill gains beyond the specific context of instruction, given that transfer of learning is considered the ultimate objective of teaching (McKeough et al., 2013). Participants who engage in the AWM curriculum exhibit evidence of transfer beyond the intervention context in which development occurred (see Table 3).

**TABLE 3:** Gains of engagement in the “Argue with Me” (AWM) curriculum and evidence of transfer.

Study	Intervention gains	Transfer of gains
Kuhn et al. (2008): US sample	Antilogos (counterargument and rebuttal) Quantity and quality of reasons (genuine justifications) Increasing meta-level usage (meta-directive) during the course of the intervention	Transfer from social topics to a novel social topic and transfer from online dialogue to individual essays
Iordanou (2010): Cyprus sample	Number and length of utterances and rebuttal strings Antilogos (counterarguments, counter-critiques)	Transfer across content domains: from social to physical science and *vice versa*
Kuhn and Crowell (2011): US sample	Antilogos (two-sided essays) Epistemological gains: greater awareness of the relevance of evidence to argument	Transfer from online dialogue to two individual essays on new social topics (two-sided essays)
Iordanou (2013): Cyprus sample	Antilogos (counterarguments and rebuttals)	Transfer from electronic dialogue to face-to-face dialogue
Kuhn et al. (2013): US sample	Epistemological gain: metatalk becomes more reciprocal, sustained with time, and focused on the argumentation process Antilogos (counterarguments)	Transfer of the two types of gains to dialogue evaluation and dialogue construction tasks
Crowell and Kuhn (2014): US sample	Antilogos (counterarguments)	Transfer from a social intervention topic to new social topics and transfer from dialogue to an evaluation task of a dialogic argumentation sequence
Iordanou and Constantinou (2014): Cyprus sample	Increase in evidence use (functional units to weaken the opponent’s claims) Increase in meta-level talk about evidence	Transfer from a physical science topic to a novel physical science topic
Iordanou and Constantinou (2015): Cyprus sample	Greater use of evidence to weaken opponents’ claims More accurate evidence and meta-level communication about evidence	Transfer from a physical science topic to a novel socioscientific topic
Kuhn and Moore (2015): US sample	More evidence-based claims New evidence integration	Transfer from dialogue to individual essays
Kuhn and Zilmer (2015): US sample	Gradual increase of metatalk statements and their acknowledgment by the opposing pair	
Iordanou (2016): Cyprus sample	Epistemological understanding	Transfer across social and science topics
Kuhn et al. (2016b): US sample	Number of functional idea units increase Antilogos (counterarguments)	Transfer across social topics and from dialogue to individual essays
Mayweg-Paus et al., 2015: US sample	Antilogos (counter-critique, counter-alternative, underminer)	
Hemberger et al. (2017): US sample	Evidence use	Transfer from dialogue to individual essays on the intervention topic and a novel topic
Papathomas and Kuhn (2017): US sample	Antilogos (counterarguments)	Transfer from dialogues with a more capable other to peer-only dialogues on a new topic
Zillmer and Kuhn (2018): US sample	Metatalk as a result of metacognitive regulation	
Iordanou et al. (2019): Cyprus and US samples	Content knowledge acquisition Acknowledging and addressing incongruent evidence	Transfer from dialogue to individual essays on intervention topic
Kuhn et al. (2019): US sample	Antilogos (counterarguments)	Transfer from dialogue to individual essays within citizenship education curriculum
Rapanta and Trovão (2019): Portuguese sample	Increased use of functional units (with reasoning fallacies related to social representations still present) Increased use of “However” compound units	Transfer from dialogue to written essays within citizenship education curriculum, two grades (seventh and 10th)
Shi (2019): Chinese sample	Use of evidence: greater use in a condition engaged in reflective activities devoted to evidence, in addition to the AWM reflective activities	Transfer from dialogue to written essays on intervention and transfer topics
Shi et al. (2019): US sample	Greater use of support own and weaken other evidence on intervention topic	Transfer from dialogue to individual essays
Iordanou and Kuhn (2020): Cyprus sample	Gains in evidence used to weaken other, weaken own, and support other	Transfer from first to second year, with a new topic in the physical science domain
Shi (2020b): Chinese sample	Metatalk grows more frequent, becoming increasingly focused on evaluating the source of evidence and better sustained over successive turns Gains in epistemological understanding (Livia task) and intellectual disposition to engage in argument and recognize its value	
Shi (2020a): Chinese sample	Counterargument and evidence use to justify claims	Transfer from dialogue to written constructed dialogue
Iordanou (in press): Cyprus sample	Greater diversity of arguments, taking multiple considerations (both social and science-related) into account Two-sided arguments	Transfer from dialogue to individual essay on intervention topic
Iordanou (submitted): Cyprus sample	More evidence used to weaken other’s position Improvement in metastrategic and epistemological awareness	
Iordanou and Fotiou (submitted): Cyprus sample	Multiple-text comprehension Use of weaken-other evidence	Transfer from dialogue to individual essays
Matos (2021): Brazilian sample	More frequent evidence-based arguments and integration of belief-incongruent statements with belief-congruent ones	Transfer from dialogue to essay on a novel topic
Rapanta (submitted): Portuguese sample	Increased use of functional units and “However” compound units	Transfer from “learning to argue” to “arguing to learn”: improvement in content-related reasoning tasks without other type of training Gains observed across four different disciplinary areas (natural science, history, language, and citizenship education)

Following engagement in dialogic argumentation in the context of the AWM curriculum, individuals’ transition from egocentric presentation of their own perspective to addressing the other side’s perspective and using counterarguments was apparent not only in peer discussion but also in students’ individual writing (Kuhn et al., 2008; Iordanou and Constantinou, 2014; Hemberger et al., 2017; Iordanou et al., 2019; Shi, 2019; Shi et al., 2019; Iordanou and Kuhn, 2020) as well as in solitary dialogues (Shi, 2020a) the participants constructed. The gains developed during practice on the social plane transferred to the individual plane, consistent with Vygotsky’s sociocultural framework of the interiorization of action from the social plan to the internal, individual, plane (Vygotsky, 1978).

Transfer of strategic gains was also observed to a novel, non-intervention topic. For example, gains in using counterarguments transferred from one physical science topic, e.g., alternative sources for producing electricity, to another physical science topic, e.g., genetically modified food (Iordanou and Kuhn, 2020), or from a social science topic, e.g., homeschooling, to another social topic, e.g., capital punishment (Kuhn et al., 2008). Transfer was also observed across knowledge domains, that is, across topics from different knowledge domains. A study by Iordanou (2010) comparing the AWM method in a physical science domain and in a social domain showed that transfer was evident in both directions; that is, students whose intervention focused on a social topic showed transfer to a post-intervention assessment of dialogic skill in a science topic and *vice versa*. However, a difference in the magnitude of transfer was observed, with only students in the science intervention condition able to transfer their skill gain to the social topic to the degree that these skills were mastered in the science topic. Thus, argumentive competence in the science domain is amenable to the same development as in the social domain, although specific engagement and practice within the science domain is warranted. The evidence for transfer across topics and generality observed is considerable, but we should not draw the conclusion that content makes no difference (Kuhn et al., 2013).

Furthermore, transfer was observed across communication modes. Many studies showed transfer from arguing with another person online *via* computer to writing, usually involving handwritten individual essays (Kuhn et al., 2013, 2019; Kuhn and Moore, 2015; Hemberger et al., 2017; Shi et al., 2019; Iordanou and Kuhn, 2020; Shi, 2020a). A study by Iordanou (2013) showed transfer from arguing on the computer to arguing face-to-face. Primary school students engaged in the AWM method *via* an instant messaging software on the computer. Although the participants initially exhibited limited ability in arguing both face to face and on the computer, by the end of the intervention, they exhibited significant advances in both modes. The gains of practice in the electronic mode—increased levels of counterargument and rebuttal—successfully transferred to the face-to-face mode.

Transfer of AWM gains was also observed in other tasks. Iordanou and Fotiou (submitted) examined the effectiveness of engagement in dialogic argumentation in relation to its ability to promote multiple-text comprehension. The multiple-text comprehension of primary school students who engaged in the AWM method was compared with that of a control group, who engaged in business-as-usual school curriculum. Only the experimental group improved in multiple-text comprehension. They showed progress in both argument skill and multiple-text integration skill throughout engagement in the intervention. Engagement in dialogic argumentation can thus serve as a promising pathway toward multiple-text comprehension. Dialogic argumentation, in which a contrasting perspective is embodied in a “real” person, as in the AWM method, may have benefited thinking about the issue, probably by emphasizing that there indeed exists a flesh-and-blood other who supports such views (Mill, 1859/1996; Iordanou and Kuhn, 2020). Recognizing that alternative positions exist on an issue, an epistemological achievement, is fundamental for multiple-text comprehension (Britt and Rouet, 2012; List and Alexander, 2019). The integration of contrasting views requires one to appreciate the need for and recognize the value of engaging in this task in order to expend the effort to engage in it, a recognition only mature epistemological understanding can provide (Kuhn, 2020).

### Explaining Transfer of Gains

The transfer of gains observed from the intervention context to novel ones shows that something is developing that, once developed, is then transferable to a context different from the one in which it has originally developed. What is developing that supports the transfer of gains in argument skills? What is the mechanism of transfer? Studies using the microgenetic method suggest that a metastrategic understanding of the norms of argumentation is developing and supports the development of argument skills (Kuhn et al., 2008, 2013; Iordanou and Constantinou, 2015; Kuhn and Zillmer, 2015; Shi, 2020b; Iordanou, submitted).

Kuhn et al. (2008) examined young adolescents’ development of better meta-level understanding about argumentive discourse and its goals. They particularly examined the claim that developing a meta-level understanding of the goals of argumentation, namely engaging in one another’s claims and undertaking to weaken them, as well as seeking acceptance of one’s own claims, what Walton (1989) identifies as the dual goal of argumentation, is likely to support progress in the procedural aspect. In an attempt to heighten such awareness, Kuhn et al. (2008) have implemented three techniques. Firstly, they ask participants who shared the same position to work in pairs in engaging in dialogues with opposing pairs, promoting planning and evaluation within the same-side pair. Secondly, they ask participants to engage in an explicit reflective activity in which they contemplate a transcript of their own previous dialogues, made possible by the record preserved by the electronic medium. Using a microgenetic method, they examined the processes of change during a year-long intervention program involving the AWM method. Observation captured both argumentive strategies within the discourse, but also metatalk, defined as talk about the discourse as opposed to talk about the topic. Participants showed significant gains in meta-level talk over the course of the intervention, in addition to gains in the use of counterargument strategies aiming to weaken others’ positions. These findings suggest that metal-level awareness and understanding of argumentation is developing through engagement in dialogic argumentation and supports development at the performance level.

Kuhn et al. (2013), further examining individuals’ meta-level understanding while engaging in the AWM method over the course of 3 years, confirmed and extended these earlier findings. Increasing metatalk revealed students becoming more explicit in their understanding of argumentation norms over time. This metatalk became more reciprocal and sometimes took a directive character, with one member of the pair providing meta-level scaffolding for the other. Shi (2020b) recently provided further support of the findings of developing meta-level understanding, reporting also an increasing prevalence of metatalk in evaluating sources of evidence.

Iordanou and Constantinou (2015); Shi (2019), and Iordanou (submitted) revealed the unique contribution of reflective activities within the AWM method in promoting individuals’ meta-level understanding of argumentation. Iordanou and Constantinou (2015) asked students to engage in reflective activities, which, in addition to prompting students to reflect on whether they constructed counterarguments and rebuttals, as was the case in the Kuhn et al. (2008) study, prompted them to reflect also on whether they had used evidence in their counterarguments or rebuttals. The results showed significant gains in primary school students’ meta-level talk as well as greater use of evidence-based counterarguments immediately after students engaged in reflective activities within the AWM method. Shi (2019) examined a group who engaged in additional reflective activities focusing on evidence, in addition to engaging in the reflective activities already a part of the AWM method that focus on the use of counterarguments and rebuttals. Compared with a group who engaged only in the reflective activities of the AWM method, Shi found that the additional reflection led to additional gains in argumentive writing, particularly in coordinating evidence and claim. Iordanou (submitted) employed an experimental design comparing two conditions: the AWM method vs. the AWM method minus its reflective activities (control condition). Participants who engaged in reflective activities related to argumentation, in addition to practice in argumentation—the AWM method—outperformed participants who only engaged in argumentation practice. The AWM group showed greater gains in argument skill—particularly in employing evidence to weaken an opposing position. Microgenetic analysis of dialogues during the interventions revealed a different pattern of progress across the two conditions. Experimental condition participants exhibited gradual and ultimately greater improvements at both the strategic and meta levels compared to the control condition participants.

This line of work shows that meta-level awareness and understanding of argumentation develop with extended practice in and reflection on argumentive discourse. Gains in meta-level awareness appear hand in hand with gains at the strategic level, suggesting a bootstrapping relation between performance at the strategic level and meta-level awareness and understanding of argumentation (Kuhn and Pearsall, 1998). The meta-level awareness and understanding that are developing involve both metastrategic knowledge, which is an understanding about how to produce, for example, a counterargument or use a piece of evidence, and epistemological understanding. The latter entails appreciation of counterargument and evidence as critical components of argumentation as well as recognition of the point of argument and value argumentation in influencing others (Kuhn and Crowell, 2011; Kuhn et al., 2013). Shi (2020b) examined Chinese middle school students’ disposition to engage in argumentive discourse after participating in a program employing the AWM method. She found that participants showed greater endorsement of argumentive discussion as a valuable activity than did peers in a non-participating control group.

Another prominent epistemological achievement observed to develop among adolescents engaged in the AWM method is the appreciation of the subjective nature of human knowledge and acknowledgment of alternative interpretations. Growing attention to others’ positions implies an implicit developing understanding that these are worthy of consideration. More direct evidence comes from an experimental study (Iordanou and Kuhn, 2020) comparing the collaborative (same-side) and adversarial (mixed-position) discourse conditions. The adversarial condition prompted more attention to evidence pertinent to the opposing position and greater gains in the use of evidence weakening the opposing position. Finally, examination of meta-level communication provides evidence of growing epistemological understanding reflected, for example, in requests to clarify whether an opponent’s claim represents a personal view or is based on evidence (Kuhn et al., 2013; Shi, 2020b). Even more direct evidence, based on explicit measures of epistemological understanding, is also available, confirming advances in epistemological understanding following engagement and practice in dialogic argumentation (Iordanou, 2010, 2016; Zavala and Kuhn, 2017; Shi, 2020b).

## Pedagogical Implications and Open Paths for Future Research and Application

The review of the literature on the dialogue-based curriculum proposed by Kuhn and her colleagues indicates that it constitutes a powerful approach to fostering dialogic skills and for extending those skills to new content and to students’ individual writing. The remarkable consistency with which the findings of the AWM curriculum have been replicated in almost 30 studies, in different countries worldwide—the United States, Europe, and Asia—since its early implementations shows that it has broad potential either as a stand-alone curriculum for developing argument skills or contextualized within different knowledge domains—social, physical science, socioscientific—and the gains have the potential to transfer to novel topics, domains, communication modes, and tasks.

According to Wilson and Bai (2010), for students to become metacognitive and therefore self-regulated learners, teachers should engage students in problem-solving activities, allowing them to share their thinking and discuss their problem solving, to generate their own questions, and to explain their answers. All of these activities are present in the AWM curriculum. In addition, the AWM method goes a step further: not only do students learn how to work independently and collaboratively, as may happen in many structured inquiry-based learning environments, but they also become epistemic learners, or in Resnick’s and colleagues’ perspective, members of a community in which each one is accountable to the other (Michaels et al., 2008; Resnick et al., 2010). This fostering of epistemic accountability is a major product of the AWM curriculum.

Although some research examining the unique contribution of particular features of the AWM curriculum has been conducted to better understand how gains are achieved, there remain many questions to be addressed to gain a fuller understanding of how the curriculum functions at both the individual and social levels, its pedagogical implications, and its full potential. There are components of the multicomponent AWM curriculum whose roles have not yet been fully examined, such as the role of same-side peer collaboration in arguing against peers holding opposing views and which forms of collaboration are most effective. The roles of group work, peers’ feedback, and visual aids, such as the use of different colored cards to represent the different components of an argument and their connection in the form of a sequence (argument–counterargument–rebuttal), remain to be established. Future research can also examine the specific challenges of different forms of evidence. Evidence in question-and-answer format has been shown to be more effective than traditional text (Iordanou et al., 2019); however, the effects of other forms of information, such as graphs, tables, and images (Iordanou and Constantinou, 2015), have yet to be examined. Also, what are the effects on the ability to evaluate evidence, as well as on inquiry skills, as different epistemic standards regarding evidence develop?

The AWM method, which centers around dense engagement in peer dialogue, has been proven sufficient with little in the way of adult instruction. Future work can compare the achievements of the AWM method to those of more explicit direct instruction, in particular with respect to the development of writing. Also, determining how the AWM method can be best integrated into traditional curricular subjects and at different developmental levels requires more work. The method has been implemented thus far largely by researchers, or teachers who worked in close collaboration with researchers. We should not take for granted that all teachers will be interested in learning and implementing this method. Some teachers feel uncomfortable not having full control of what goes on in their classrooms (such as talk between students that they do not hear). Some may not be convinced that student-to-student talk is productive. Therefore, future work should explore methods of professional development of teachers that enable them to try methods that may fall outside of their present comfort zones. The connection found between teachers’ own argument skills and their facility in supporting students’ argument skill development (Lytzerinou and Iordanou, 2020), as well as evidence of gains in pre-service teachers’ argument skills following engagement in the AWM method (Iordanou and Constantinou, 2014), suggests use of the AWM method itself in the professional development of teachers. Whether such experience is sufficient to transfer to their teaching practice remains to be seen.

Another avenue for future research is to study how the curriculum can be adapted to different cultural contexts. The AWM method has been implemented with success in Eastern as well as Western cultures (Shi, 2019), with consistent findings. Yet, the question remains open of the extent to which the method will yield the same findings across a wider range of socioeconomic and academic backgrounds in non-western cultures. Even more importantly, future research is needed to investigate how the method can be adapted in order to be more suited to different cultural settings. Which are those components of the method that can or should be adapted in different cultural settings and which are essential to its effectiveness and therefore should constitute an integral part of the method across contexts? The two, Kuhn (2019) has claimed in this category, are deep engagement with a topic and dense peer-to-peer discourse.

Finally, future research can examine the transfer of gains fostered by the AWM curriculum to real-life settings and its potential to affect not only thinking but also behavior. Do the gains of the AWM curriculum transfer to whole-class discussion and to discussions, as well as individual thinking, outside of the classroom? Although there is evidence that the AWM curriculum fosters gains in intellectual values and epistemological understanding, as assessed in paper-and-pencil measures, its potential to support critical thinking in real-life contexts needs further investigation. According to Halpern (1998), “a critical thinker exhibits the following dispositions or attitudes: (a) willingness to engage in and persist at a complex task, (b) habitual use of plans and the suppression of impulsive activity, (c) flexibility or open-mindedness, (d) willingness to abandon non-productive strategies in an attempt to self-correct, and (e) an awareness of the social realities that need to be overcome (such as the need to seek consensus or compromise) so that thoughts can become actions” (p. 452). Although the connection between AWM gains and critical argumentation is clear, such gains can be further contextualized to everyday real-life decision-making. For example, does engagement in the AWM curriculum on pressing topics, such as climate change, affect individuals’ immediate or longer-term attitudes and decisions on this issue? Unlike traditional school curriculum, the AWM curriculum focuses on deep engagement with contemporary social issues, such as immigration, an engagement that prepares students for engaged, active citizenship (Kuhn et al., 2019; Rapanta and Trovão, 2019). Yet, the question of whether and how engagement in the AWM method can support more responsible citizenship is an open one. In addition to more engaged, responsible citizenship, can the curriculum promote or change fundamental values, in particular the intellectual value of appreciation for the power of argument and evidence in resolving differences? Only future research can explore the full potential of the AWM curriculum and how it can further be developed to promote among the next generation the valuing of dialogue within and across societies.

## Author Contributions

Both authors listed have made a substantial, direct and intellectual contribution to the work, and approved it for publication.

## Conflict of Interest

The authors declare that the research was conducted in the absence of any commercial or financial relationships that could be construed as a potential conflict of interest.

## Footnotes

1 This openness to also consider personally known evidence in functional units’ construction is highly important as it leaves space for pre-argumentation forms to emerge and develop throughout school-based practice. Examples of children’s pre-argumentation strategies can be found in Bova and Arcidiacono (2014) and in Arcidiacono and Bova (2015).

2 In Kuhn’s approach, argumentative writing is highly dialogic. This is why we opt for including it in the section titled “Participating in Argumentative Discourse.” Similar to academic writing, writing an argumentative essay is like entering a room in which discussion on a debatable topic has already started (Graff and Birkenstein, 2010), and therefore, it is the writer’s responsibility to be relevant to this previous and ongoing discussion.
